# miR-199a-3p inhibits cell proliferation and induces apoptosis by targeting YAP1, suppressing Jagged1-Notch signaling in human hepatocellular carcinoma

**DOI:** 10.1186/s12929-016-0295-7

**Published:** 2016-11-10

**Authors:** Kewei Ren, Tengfei Li, Wenzhe Zhang, Jianzhuang Ren, Zhen Li, Gang Wu

**Affiliations:** 1 Department of Radiology, The First Affiliated Hospital of Zhengzhou University, No. 1 Jianshe East Road, Zhengzhou, 450052 Henan People’s Republic of China; 2 Interventional Institute of Zhengzhou University, Zhengzhou, 450052 People’s Republic of China; 3 Interventional Treatment and Clinical Research Center of Henan Province, Zhengzhou, 450052 People’s Republic of China

**Keywords:** Hepatocellular carcinoma, miR-199a-3p, Yes associated protein 1, Jagged1, Notch signaling

## Abstract

**Background:**

miR-199a-3p was significantly downregulated in the majority of human hepatocellular carcinoma (HCC) tissues and HCC cell lines. Yes associated protein 1 (YAP1) was overexpressed in human HCC, which promoted HCC development and progression by upregulating Jagged1 and activating the Notch pathway. We searched potential targets of miR-199a-3p with DIANA, TargetScan and PicTar tools, and found that YAP1 is one of the potential targets. Based on these findings, we speculated that miR-199a-3p might suppress HCC growth by targeting YAP1, downregulating Jagged1 and suppressing the Notch pathway.

**Results:**

We determined the expression of miR-199a-3p and YAP1 by quantitative Real-Time PCR (qRT-PCR) and western blot assays, respectively, and found downregulation of miR-199a-3p and upregulation of YAP1 in HCC cell lines. Cell proliferation and apoptosis assays showed that miR-199a-3p suppresses HCC cell proliferation and promotes apoptosis, and knockdown of YAP1 has similar role. Furthermore, we verified that miR-199a-3p can directly target YAP1. We further investigated and confirmed that miR-199a-3p and YAP1 regulate HCC cell proliferation and apoptosis through Jagged1-Notch signaling.

**Conclusion:**

miR-199a-3p targets YAP1, downregulates Jagged1 and suppresses the Notch signaling to inhibit HCC cell proliferation and promote apoptosis. These findings provide new insights into the mechanism by which miR-199a-3p suppresses HCC cell proliferation and induces apoptosis.

## Background

Hepatocellular carcinoma (HCC) is one of the most common malignant tumor in the word, particularly in East Asia and South Africa [1, 2]. There are over 250,000 new HCC cases and an estimated 600,000 HCC deaths each year [3]. Chronic hepatitis B Virus (HBV), hepatitis C Virus (HCV) infection, and aflatoxin B1 exposure are the predominant risk factors for the initiation of HCC [4]. Although great improvements in treatment options have been achieved in the recent years, the prognosis of HCC patients remains very poor, with a 5-year survival rate about 30 % [5]. The main two reasons of the poor prognosis are the delay in diagnosis of HCC and lack of effective treatment for advanced HCC [6]. Undoubtedly, a better understanding of the underlying molecular mechanisms of the initiation and development of HCC will be conducive to identify novel biomarkers and develop effective treatment strategies, which is very significant to HCC patients. As the genesis and progress of other cancers, the initiation and development of HCC is also related to the accumulated genetic alterations [7].

MicroRNAs (miRNAs), a class of short, non-coding RNAs of about 19–25 nucleotides, post-transcriptionally regulate gene expression by binding to partially complementary sites in the 3' untranslated regions (3’UTR) of targeted mRNAs, thereby causing translational repression or messenger RNA (mRNA) degradation [8]. miRNAs are involved in various biological processes, including cell differentiation, proliferation, aging, apoptosis, migration, invasion, development and signal transduction [9]. Increasing evidence shows that there exist causal relationship between the deregulation of miRNA expression and the initiation and development of cancer, and miRNAs can play oncogenic or tumor suppressive roles in human cancers depending on the target genes [10]. In fact, many dysregulated miRNAs have been reported to play important roles in the occurrence and progression of HCC, and miRNAs have been suggested as potential biomarkers and novel therapeutic targets for HCC [11, 12].

Recently, miR-199a-3p, a cancer-associated miRNA, is widely reported to be deregulated in many malignant tumors and its role in tumor development is controversial. It can acts as either a tumor suppressor with downregulated expression in some types of cancers, such as renal cancer and bladder cancer, or an oncogene with upregulated expression in gastric cancer and colorectal cancer [13–15]. In HCC, miR-199a-3p has been reported to be downregulated compared to corresponding nontumor liver tissues [16–19]. We used DIANA, TargetScan and and PicTar to perform target prediction analysis, and found that Yes associated protein 1 (YAP1) is a potential target of miR-199a-3p. YAP1 as an oncogene is highly expressed in the various types of cancer, including HCC [20–24]. Dong et al. [24] reported that liver-specific overexpression of YAP1 leads to a greater than 5-fold size enlargement which is reversible after cessation of YAP1 expression. Recently, YAP1 has been reported to promote HCC development and progression by upregulating Jagged1 and activating the Notch pathway [25]. Therefore, we speculated that miR-199a-3p might regulate HCC cell proliferation and apoptosis in part by targeting YAP1, downregulating Jagged1 and suppressing the Notch pathway. In this study, we investigated whether miR-199a-3p targets YAP1 to downregulate Jagged1 and inhibit the Notch pathway, thereby regulating HCC cell proliferation and apoptosis.

## Methods

### Cell culture

Five human HCC cell lines (MHCC97H, Hep3B, SMMC-7721, Huh7, and HepG2) and a normal liver cell line (HL-7702) were purchased from American Type Culture Collection (ATCC, Manassas, VA, USA). Cells were cultured in Dulbecco’s modified Eagle’s medium (Invitrogen, Carlsbad, CA, USA) supplemented with 10 % fetal bovine serum (FBS, Invitrogen), 100 U/ml penicillin and 100 mg/ml streptomycin at 37 °C with 5 % CO_2_ and 95 % humidity.

### Cell treatment

Huh7 cells were transfected with miR-199a-3p mimic, small interfering RNA for YAP1 (si-YAP1), pcDNA3.1 vectors containing the cDNA of YAP1 (pcDNA-YAP1), pcDNA-Jagged1, si-Jagged1 and their respective controls (Ribobio, Guangzhou, China) by using Lipofactamine 2000 (Invitrogen) according to the manufacturer’s instructions. To investigate whether introduction of miR-199a-3p could increase HCC cell sensitivity to the drug γ-secretase inhibitor n-[n-(3,5-difluorophenacetyl-l-alanyl)]-s-phenylglycinet-butyl ester (DAPT; Calbiochem, San Diego, CA, USA), Huh7 cells transfected with miR-control or miR-199a-3p mimic were treated with DAPT at a final concentration of 10 μM for 48 h.

### MTT assay

Cell proliferation was measured using the 3-[4,5-dimethyl-2-thiazolyl]-2,5-diphenyl-2H-tetrazolium bromide (MTT; Sigma-Aldrich, St. Louis, MO, USA) assay. Briefly, cells were seeded into 96-well plates at a density of 2 × 10^3^ cells/well, and cultured for 48 h. Then 20 μl of MTT (5 mg/ml) was added in each well followed by incubation for 4 h at 37 °C. After removing the culture medium, 150 μl/well of DMSO (Sigma-Aldrich) was added to dissolve the precipitate. The absorbance was measured at 570 nm by using a plate reader. Each experiment was performed at least in triplicate.

### Flow cytometry assay

Cell apoptosis was determined by using the Annexin V-FITC apoptosis Detection Kit (BD Pharmingen, San Diego, CA, USA) according to the manufacturer’s instructions. Cells were collected, washed and stained using Annexin V-FITC and propidium iodide (PI). After incubation for 15 min in darkness, the cells were examined by flow cytometry (BD Biosciences, San Jose, CA, USA).

### Caspase 3/7 activation assay

Caspase 3/7 activity was measured using Caspase-Glo 3/7 kit (Promega, Madison, WI, USA) according to the manufacturer’s instruction.

### Real-time quantitative reverse transcription-PCR (qRT-PCR)

Total RNA was extracted from the cells using TRIzol (Invitrogen) according to the instructions of the manufacturer. The cDNA was synthesized by using SuperScript® III Reverse Transcriptase (Invitrogen), and Real-time PCR amplifications were undertaken using SYBR PrimeScript RT-PCR kit (Takara, Dalian, China). GAPDH was used as an endogenous control for mRNA. The miRNA was converted to cDNA by using TaqMan MicroRNA Reverse Transcription Kit (Applied Biosystems, Foster City, CA, USA). The qRT-PCR was performed using TaqMan Human MiRNA Assay Kit (Applied Biosystems) with normalization to U6 snRNA level. The primer sequences used were as follows: miR-199a-3p, reverse transcription 5′-GTC GTA TCC AGT GCA GGG TCC GAG GTA TTC GCA CTG GAT ACG ACT AAC CA-3′, forward 5′-GCG GCG GAC AGT AGT CTG CAC-3′ and reverse 5′-ATC CAG TGC AGG GTC CGA GG-3′; YAP1, forward 5′-CCT GCG TAG CCA GTT ACC AA-3′ and reverse 5′-CCA TCT CAT CCA CAC TGT TC-3′; GAPDH, forward 5′-GGC AAA TTC AAC GGC ACA-3′ and reverse 5′-GTT AGT GGG GTC TCG CTC CTG-3′; U6, forward 5′-CTC GCT TCG GCA GCA CA-3′ and reverse 5′-AAC GCT TCA CGA ATT TGC GT-3′.

### Western blot

Protein samples were isolated from cell lysates and the concentration of total protein was measured using BCA Protein Assay Kit (Pierce, Rockford, IL, USA). Equal amounts of samples (50 μg/lane) were separated by 10 % SDS-PAGE and then transferred onto a polyvinylidene difluoride membrane. After blocking in 5 % skimmed milk, the membrane was incubated with the specific antibody. The following primary antibodies were used: YAP1 (Cell Signaling, Danvers, MA, USA), Jagged1 (Santa Cruz Biotechnology, Santa Cruz, CA, USA), Notch intracellular domain (NICD; Santa Cruz Biotechnology), Hes-1 (Abcam, Cambridge, UK), and β-actin (Sigma-Aldrich). Goat anti-mouse/rabbit IgG antibody conjugated to horseradish peroxidase (HRP) was used as the secondary antibody (Gene Tech, Shanghai, China). The blotted proteins were determined by using enhanced chemiluminescence regents (Thermo Scientific, Waltham, MA, USA).

### Luciferase reporter assay

The wild-type 3’UTR (3’UTR-WT) of YAP1 containing the miR-199a-3p binding sites was obtained by PCR. Mutant YAP1 3’UTR (3’UTR-MUT) which the mutations occur in the conserved binding sites for miR-199a-3p, was generated by using overlapping extension PCR. The fragment of YAP1 3’UTR-WTand the mutant 3’UTR fragment were inserted into XhoI/NotI-digested psiCHECK-2 vector (Promega) containing firefly and renilla luciferase reporter gene, respectively. The sequence of 3’UTR-WT of YAP1 included the putative binding sites of miR-199a-3p, while the sequence of 3’UTR-MUT of YAP1 not. Then the psiCHECK-2 vectors with 3’UTR-WT or 3’UTR-MUT regions of YAP1 were transfected into Huh7 cells containing miR-199a-3p mimics or miR-control, respectively. At 48 h after the transfection, cells were collected and lysed for luciferase detection using Dual-Luciferase Reporter System (Promega).

### Statistical analysis

The SPSS 15.0 software (SPSS, Chicago, Illinois, USA) was used for all statistical analyses. All data were expressed as mean ± standard error (SE). Statistical analyses were determined by using two-tailed student’s *t*-test or analysis of variance (ANOVA). *P* < 0.05 was considered statistically significant.

## Results

### miR-199a-3p was downregulated and YAP1 was upregulated in HCC cell lines

**Fig. 1:**
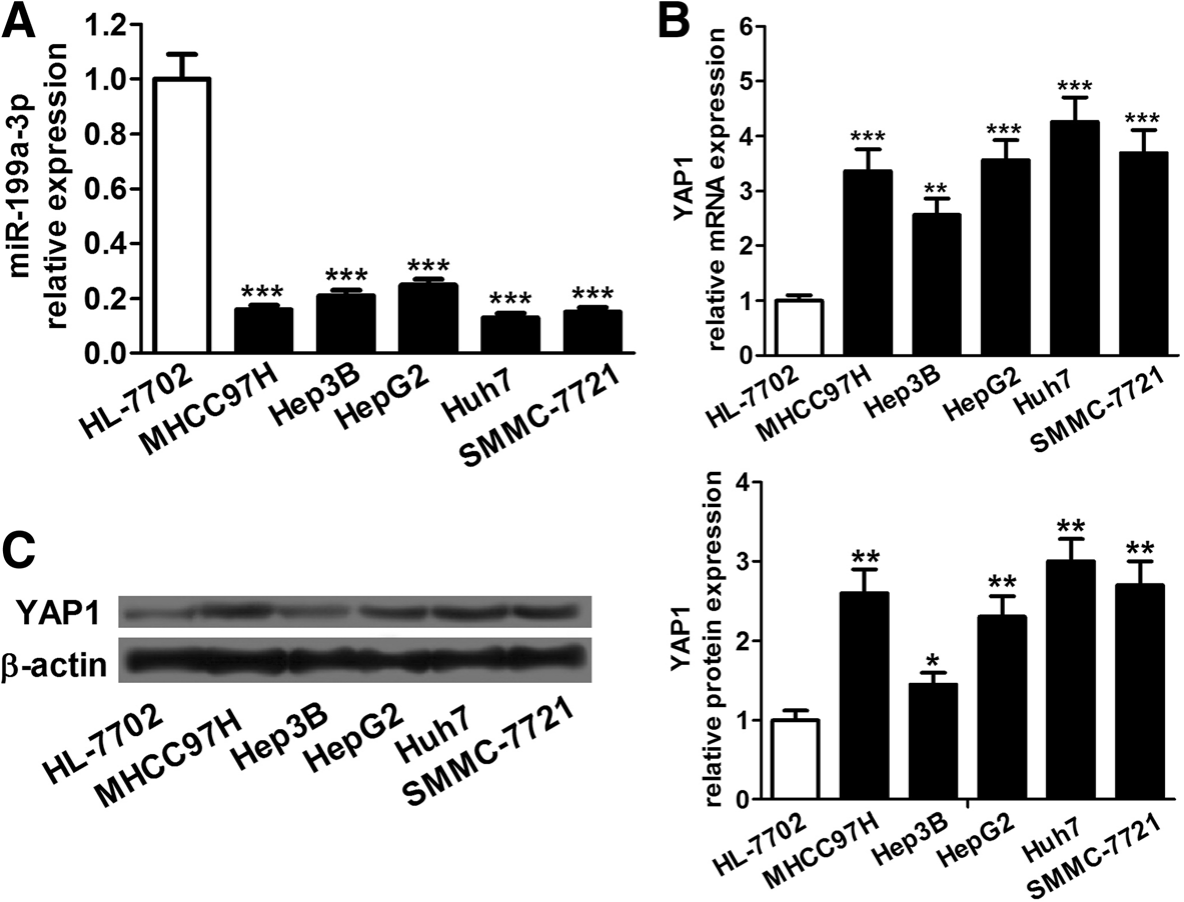
The expression levels of miR-199a-3p and YAP1 in HCC cell lines. **a** Comparing differences in the expression levels of miR-199a-3p between HCC cell lines and normal liver cell line HL-7702. **b** and **c** The expression levels of YAP1 mRNA and protein are determined by qRT-PCR and western blot. *P < 0.05, **P < 0.01 and ***P < 0.001

miR-199a-3p expression was determined in five human HCC cell lines (MHCC97H, Hep3B, Huh7, SMMC-7721 and HepG2) and a normal liver cell line (HL-7702) by qRT-PCR. The results showed that the expression of miR-199a-3p in HCC cell lines was very lower than that in HL-7702 cells (Fig. 1a). We also detected the expression levels of YAP1 mRNA and protein in HCC cell lines and HL-7702 cells using qRT-PCR and western blot, respectively. When normalized to the normal liver cell line, the expression of YAP1 mRNA and protein were significantly increased in HCC cell lines (Fig. 1b and c). These data indicated that miR-199a-3p was downregulated and YAP1 was upregulated in HCC cell lines.

### Overexpression of miR-199a-3p and knockdown of YAP1 inhibited proliferation and promoted apoptosis in HCC cells

**Fig. 2:**
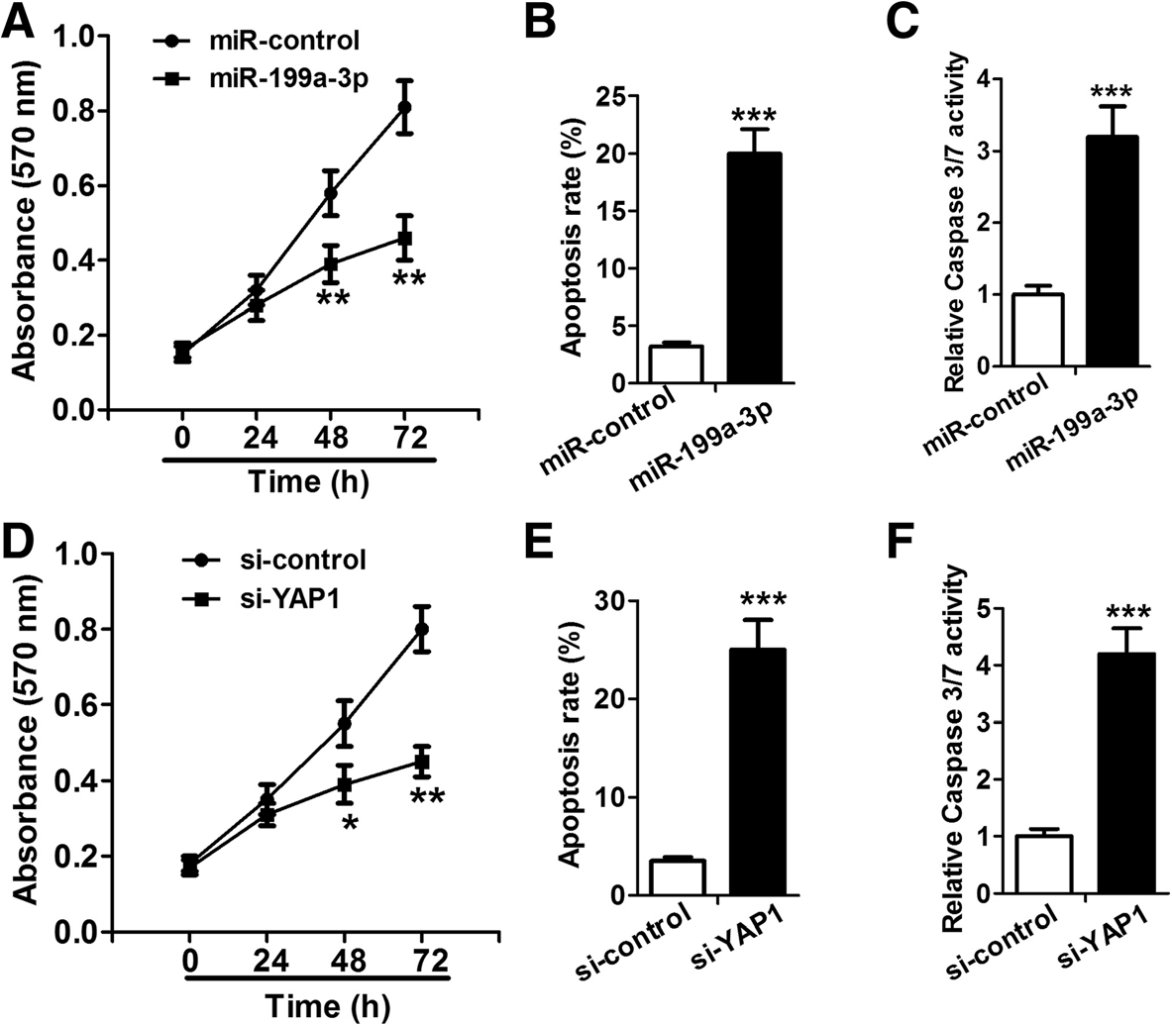
Overexpression of miR-199a-3p and knockdown of YAP1 inhibit proliferation and induce apoptosis in HCC cells. **a** and **d** The effects of overexpression of miR-199a-3p and knockdown of YAP1 on Huh7 cell proliferation are detected by MTT assay. **b** and **e** The effects of overexpression of miR-199a-3p and knockdown of YAP1 on Huh7 cell apoptosis are assessed by flow cytometry assay. **c** and **f** Caspase 3/7 activity is measured in Huh7 cells transfected with miR-control, miR-199a-3p mimic, si-control or si-YAP1. *P < 0.05, **P < 0.01 and ***P < 0.001

In view of downregulation of miR-199a-3p and upregulation of YAP1 in HCC cells, we further investigated whether overexpression of miR-199a-3p and knockdown of YAP1 had the same effects on proliferation and apoptosis of HCC cells. We transfected Huh7 cells with miR-199a-3p mimic and mimic control, respectively. As measured by MTT assay, upregulation of miR-199a-3p led to an obvious decrease of cell proliferation rate in HCC cells (Fig. 2a). Flow cytometry assay showed that apoptosis rate increased more than 5-fold in Huh7 cells transfected with miR-199a-3p mimic, compared to control group (Fig. 2b). Caspase 3/7 activation assay showed that Caspase 3/7 activity increased more than 2-fold in the cells transfected with miR-199a-3p mimic, compared to control group (Fig. 2c). In addition, to understand the effects of YAP1 on HCC cells, we transfected Huh7 cells with small interfering RNA for YAP1 (si-YAP1) and si-control, and performed MTT, flow cytometry and Caspase 3/7 activation assays. As expected, YAP1 knockdown obviously suppressed proliferation and induced apoptosis in Huh7 cells (Fig. 2d–f). Thus, upregulation of miR-199a-3p and YAP1 deletion had the same effects on proliferation and apoptosis of HCC cells, which inhibited HCC cell proliferation and promoted apoptosis.

### YAP1 was identified as a functional target of miR-199a-3p

**Fig. 3:**
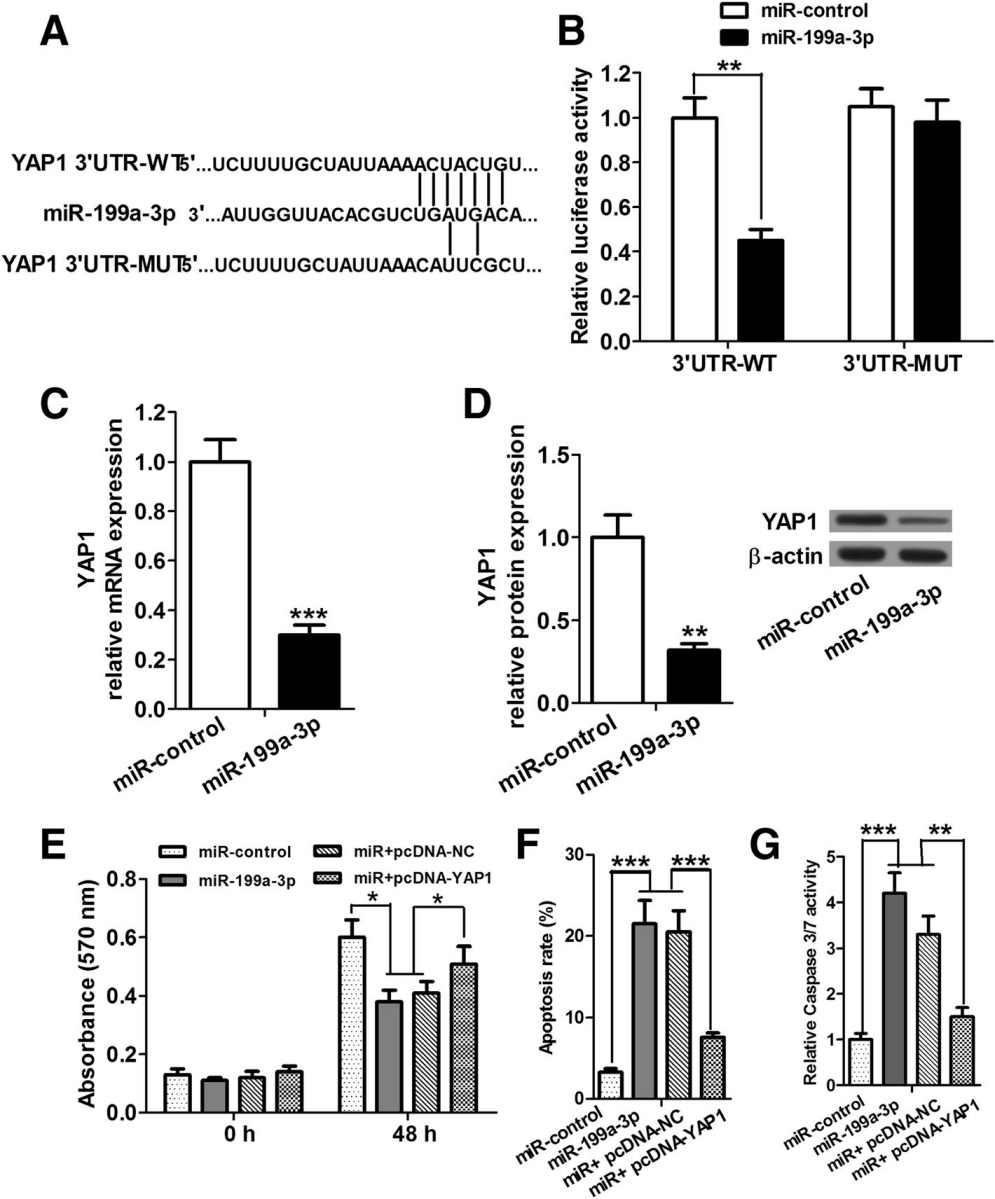
YAP1 is a functional target of miR-199a-3p in HCC cells. **a** Bioinformatics-based target prediction analysis represents that YAP1 is a potential target of miR-199a-3p and the putative binding sequence is in the 3’-UTR of YAP1 mRNA. **b** Luciferase reporter assay shows that the luciferase activity in cells containing miR-199a-3p and 3’UTR-WT of YAP1 is markedly decreased compared with the negative control and 3’UTR-MUT groups. **c** and **d** qRT-PCR and western blot analyses shows that miR-199a-3p overexpression signigficantly downregulates mRNA and protein levels of YAP1.**e**–**g** MTT, flow cytometry and Caspase 3/7 activation assays show that the upregulation of YAP1 partially reverses miR-199a-3p’s effect on the proliferation and apoptosis of Huh7 cells. *P < 0.05, **P < 0.01 and ***P < 0.001

To further investigate the molecular mechanism by which miR-199a-3p inhibited proliferation and promoted apoptosis in HCC cells, publicly available databases (DIANA, TargetScan and PicTar) were searched for the targets of miR-199a-3p, and YAP1 was a potential target of miR-199a-3p based on the putative target sequence at 249–255 base pairs of the YAP1 3’UTR (Fig. 3a). We also performed Gene Ontology (GO) analysis (data not shown), and found YAP1 is involved in various biological processes, such as cellular macromolecule metabolic process, developmental process, regulation of signaling, cell proliferation and apoptosis. Then luciferase reporter assay was performed on Huh7 cells to identify whether miR-199a-3p directly interacted with the 3’UTR of YAP1 mRNA. The results showed that overexpression of miR-199a-3p reduced the luciferase activity of the luciferase reporter containing wild type 3’UTR of YAP1 but not mutant reporter (3’UTR-MUT) vector (the sequences of YAP1 3’UTR-WT/MUT shown in Fig. 3a), which indicated that YAP1 is indeed a direct target of miR-199a-3p (Fig. 3b). Furthermore, to confirm that miR-199a-3p can regulate YAP1 expression, qRT-PCR and western blot assays were performed to determine the expression of YAP1 mRNA and protein in response to the alteration of miR-199a-3p expression in Huh7 cells. Results from qRT-PCR revealed that miR-199a-3p markedly reduced the expression of YAP1 mRNA compared with the control group (Fig. 3c). Western blot assay showed an obvious reduction of YAP1 expression after overexpression of miR-199a-3p (Fig. 3d). Proliferation (MTT) and apoptosis (flow cytometry and Caspase 3/7 activation) assays showed that the upregulation of YAP1 partially reversed miR-199a-3p's effect on the proliferation and apoptosis of Huh7 cells (Fig. 3e–g). While overexpression of YAP1 fails to fully restore miR-199a-3p's effect on proliferation and apoptosis, probably due to a sufficiently high endogenous level of YAP1 already existing in Huh7 cells. Taken together, our data strongly suggest that YAP1 is a target of miR-199a-3p, and miR-199a-3p regulates proliferation and apoptosis of HCC cells, at least partially, by directly targeting YAP1.

### Jagged1 relieved the effects of upregulation of miR-199a-3p and inhibition of YAP1 on HCC cells

**Fig. 4:**
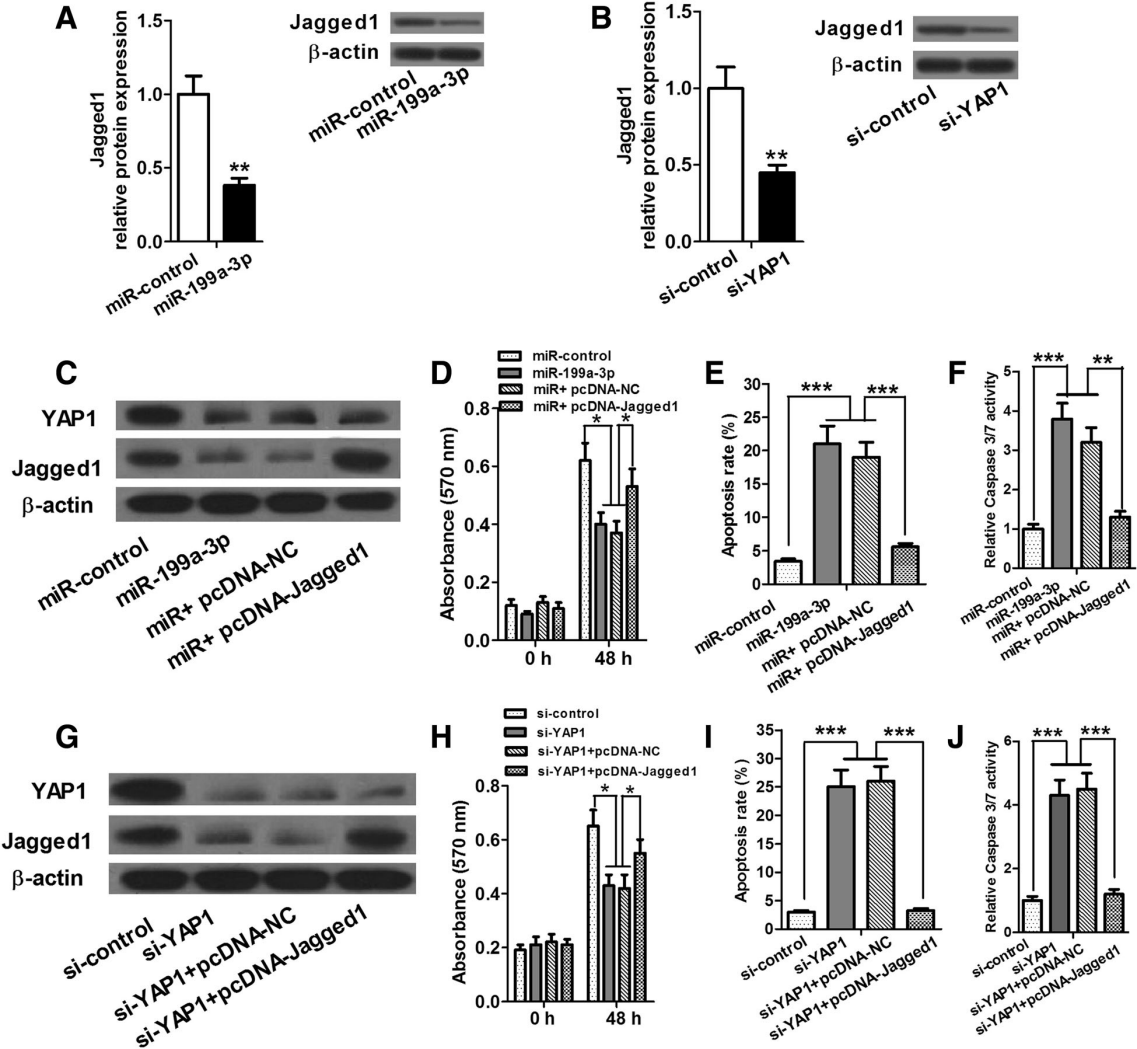
The Notch ligand Jagged1 is a functional YAP1 target. **a** and **b** High expression of miR-199a-3p and YAP1 silencing significantly inhibit protein level of Jagged1. **c** and **g** Wetern blot assays confirm that transfection is successful for all combinations. **d**–**f** Overexpression of miR-199a-3p reduces cell proliferation and induces apoptosis, however, Jagged1 relieves these effects in Huh7 cells. **h**–**j** si-YAP1 significantly inhibits cell proliferation and induces apoptosis in Huh7 cells, while Jagged1 reverses these effects. *P < 0.05, **P < 0.01 and ***P < 0.001

To investigate whether miR-199a-3p targets YAP1, and downregulates Jagged1 to inhibit proliferation and promote apoptosis of HCC cells, we firstly upregulated miR-199a-3p and downregulated YAP1 by transfecting miR-199a-3p mimics, si-YAP1 and their respective controls into Huh7 cells, respectively, and observed the expression of Jagged1 protein. Western blot analysis presented that efficient upregulation of miR-199a-3p and inhibition of YAP1 reduced expression of Jagged1 protein (Fig. 4a and b). In view the inhibitory effects of miR-199a-3p and YAP1 knockdown on Jagged1 expression in Huh7 cells, we further investigated whether miR-199a-3p and YAP1 silencing had the same effects on the function of Jagged1. First, we transfected Huh7 cells with following reagents: miR-control, miR-199a-3p mimic, miR-199a-3p mimic + pcDNA-NC (miR + pcDNA-NC), miR + pcDNA-Jagged1, si-control, si-YAP1, si-YAP1 + pcDNA-NC, si-YAP1 + pcDNA-Jagged1. Wetern blot assays confirmed that transfection was successful for all combinations (Fig. 4c and g). Then we performed proliferation and apoptosis assay. Proliferation assay showed that high expression of miR-199a-3p and knockdown of YAP1 significantly reduced proliferation in Huh7 cells at 48 h, while the proliferation rate in pcDNA-Jagged1 co-transfected cells was obviously increased (Fig. 4d and h). The examination of apoptosis showed that apoptosis was induced in Huh7 cells with miR-199a-3p overexpression or YAP1 deletion, inversely, less apoptosis was observed after cells were co-transfected with pcDNA-Jagged1 vector (Fig. 4e, f, i and j). These data indicated that upregulation of miR-199a-3p and inhibition of YAP1 suppressed cell proliferation and promoted apoptosis, whereas, Jagged1 could relieve these effects on HCC cells.

### miR-199a-3p and YAP1 regulated proliferation and apoptosis of HCC cells through Jagged1-Notch signaling

**Fig. 5:**
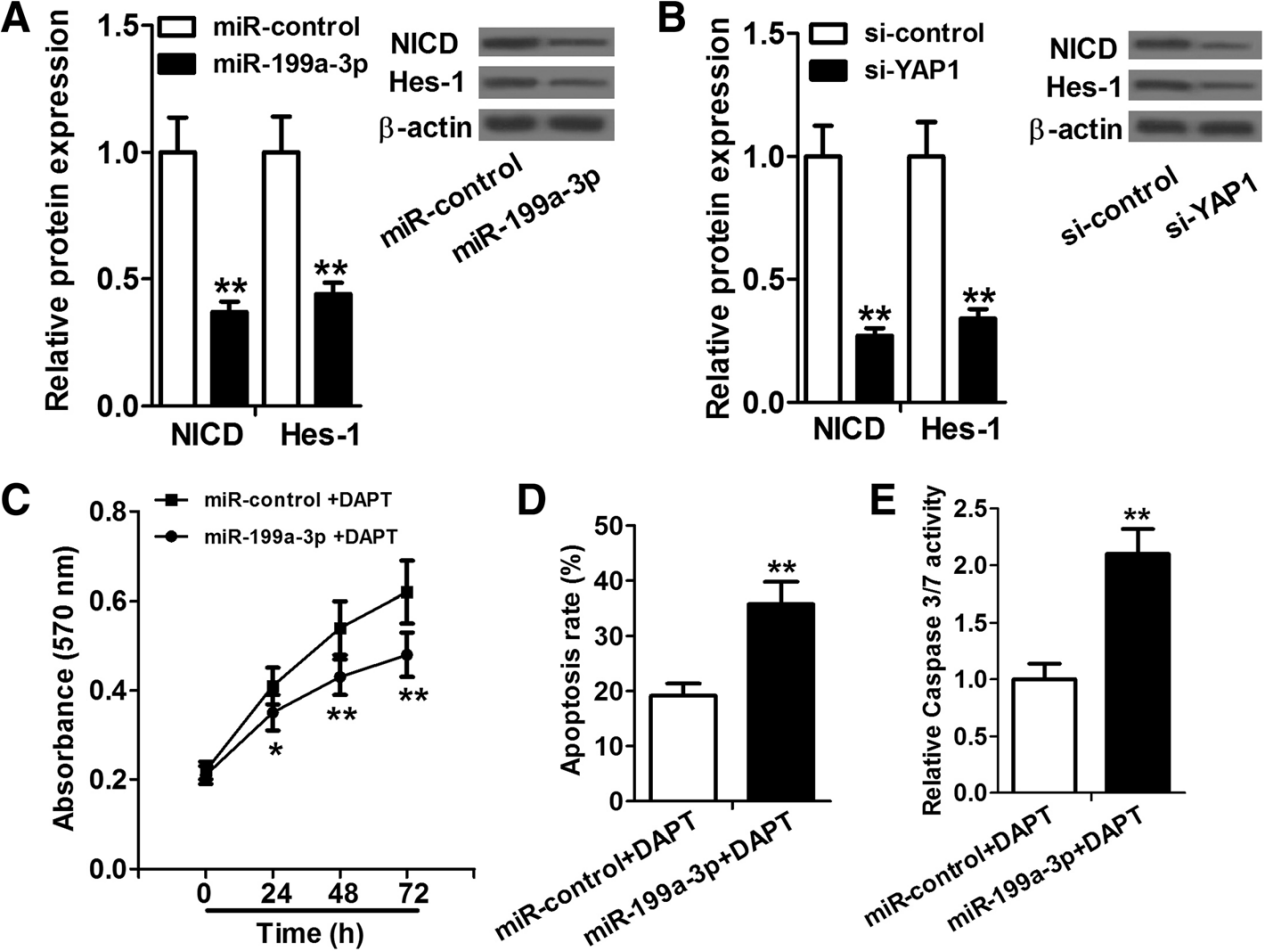
miR-199a-3p and YAP1 regulated proliferation and apoptosis of HCC cells through Jagged1-Notch signaling. **a** and **b** Huh7 cells transfected with miR-199a-3p mimics and si-YAP1 show signigficantly lower protein levels of NICD and Hes-1 than control cells. **c**–**e** Huh7 cells treated by miR-199a-3p mimic and DAPT have an obviously lower cell proliferation rate and a significantly higher apoptosis rate than the cells treated by miR-control and DAPT. *P < 0.05 and **P < 0.01

Jagged1 as a Notch ligand is an important component of the Notch pathway. We further determined the effects of miR-199a-3p and YAP1 on the Notch signaling. Western blot analysis showed that miR-199a-3p overexpression and YAP1 knockdown in Huh7 cells reduced expression of Notch intracellular domain (NICD) and the Notch target gene Hes-1, indicating diminished the Notch pathway activity (Fig. 5a and b). In addition, we investigated whether introduction of miR-199a-3p could increase the drug sensitivity of DAPT-treated HCC cells. DAPT, a γ-secretase inhibitor, can block the Notch signaling. The data suggested that Huh7 cells treated by miR-199a-3p mimic and DAPT had an obviously lower cell proliferation rate and a significantly higher apoptosis rate than the control cells treated by miR-control and DAPT, which indicated that miR-199a-3p could increase HCC cell sensitivity to DAPT (Fig. 5c–e). Taken together, our results, for the first time, illustrated the miR-199a-3p-YAP1-Jagged1-Notch signaling in HCC, in which miR-199a-3p targets YAP1 and regulates the Jagged1-Notch signaling to inhibit the tumorigenesis of HCC. This regulatory network may help to lead a better understanding of HCC initiation and progression and contribute to the miRNA-targeted therapy for this deadly disease.

## Discussion

Primary liver cancer is the third leading cause of cancer-related death in the world, and HCC is the most frequent subtype, which has a complex pathogenesis [26]. Although the major risk factors are known, therapeutic options in HCC patients remain limited, which less than 30 % of newly diagnosed HCC patients are eligible for currently curative therapies such as liver transplantation, resection or local ablation [27]. This is in part because of our incomplete understanding of the cellular and molecular mechanisms of HCC initiation and progression. Therefore, deepening understanding of the cellular and molecular mechanisms influencing HCC development is very essential. With an increasing number of high-throughput data from different genomic levels being generated, the possibility of integrating these data provides us an excellent opportunity to identify critical genetic and epigenetic alterations in HCC. Among them, miRNAs and their targets may represent potential novel biomarkers or therapeutic targets for HCC. Many studies have demonstrated that miRNAs modulate the expression of tumor suppressor or oncogene, which suggests a new mechanism involved in the initiation and progression of HCC.

miR-199a-3p was of interest to us because it was downregulated in several HCC studies [18, 28]. miR-199a-3p was also decreased in alcohol induced steatohepatitis, a known precursor to HCC [29]. Restoration of miR-199a-3p decreased HCC cell invasion and proliferation [30]. By bioinformatics-based target prediction analysis with DIANA, TargetScan and PicTar tools, we found that CD44, PAK4 and YAP1 were potential targets of miR-199a-3p. Henry et al. [19] reported that miR-199a-3p represses proliferation of CD44 positive HCC cell lines by targeting CD44. Hou et al. [16] reported that miR-199a/b-3p target PAK4 to inhibit the growth of HCC through suppressing PAK4/Raf/MEK/ERK pathway. YAP1 was upregulated in HCC tissues and cells, and promoted HCC development and progression [24]. In addition, recently miR-497 and miR-506 were reported to inhibit cell proliferation and induce apoptosis by targeting YAP1 in HCC [31, 32]. Therefore, we speculated that miR-199a-3p might target YAP1 to regulate HCC cell proliferation and apoptosis. Our experimental results confirmed our speculation that downregulation of miR-199a-3p and upregulation of YAP1 existed in HCC cells, and miR-199a-3p inhibited cell proliferation and induced apoptosis partly by targeting YAP1.

The Notch pathway is an evolutionarily conserved signaling, which includes Notch ligands, receptors, target transcription factors, and negative and positive modifiers. As an important signaling pathway, Notch pathway not only participates in embryonic development and cell fate determination, but also plays an important role in cancer [33]. However, the role of Notch signaling in tumorigenesis is controversial. For instance, the Notch pathway is aberrantly activated in some malignant tumors, such as colorectal, breast and lung carcinomas, and play the tumor-facilitative role, which the inappropriate activation of Notch in these tumors is caused by a dysbalance between Notch ligands, such as Jagged1 and inhibitors [34, 35]. While activation of the Notch pathway has also been reported to suppress tumor growth, including neural crest tumors and skin cancer [36, 37]. The Notch pathway seems to act as a tumor suppressor or an oncogene, depending on the tissue type. For the role of the Notch pathway in HCC, many studies have demonstrated that the Notch signaling pathway is activated, and plays the tumor-facilitative role in HCC [38, 39]. Recently, Jagged1-Notch signaling was reported to be activated by YAP1, thereby promoting HCC development and progression [25]. Based on these findings, we speculated that miR-199a-3p might regulate HCC cell proliferation and apoptosis in part by targeting YAP1 and suppressing Jagged1-Notch signaling. Our experiments also confirmed our speculation that miR-199a-3p inhibits HCC cell proliferation and induces apoptosis in part by targeting YAP1 and suppressing Jagged1-Notch signaling.

## Conclusions

In summary, our results, for the first time, illustrated the miR-199a-3p-YAP1-Jagged1-Notch signaling in HCC, in which miR-199a-3p targets YAP1 and represses the Jagged1-Notch signaling to inhibit the tumorigenesis of HCC. This regulatory network contributes to a better understanding of HCC initiation and progression and the miRNA-targeted therapy for this deadly disease.

## Abbreviations

3'UTR: 3'-untranslated region
HCC: Hepatocellular carcinoma
miRNA: MicroRNA
qRT-PCR: Real-time quantitative reverse transcription-PCR.
YAP1: Yes associated protein 1
